# Temporal Trends and Practice Patterns Associated With Utilization of Catheter-Based Interventions for Pulmonary Embolism

**DOI:** 10.1016/j.jscai.2025.103736

**Published:** 2025-07-23

**Authors:** Nathan W. Watson, Michael R. Jaff, Brett J. Carroll, Hibiki Orui, Siling Li, Yang Song, Jeffrey L. Weinstein, Robert W. Yeh, Eric A. Secemsky

**Affiliations:** a Department of Medicine, Brigham and Women’s Hospital, Harvard Medical School, Boston, Massachusetts; b Richard A. and Susan F. Smith Center for Outcomes Research in Cardiology, Department of Medicine, Beth Israel Deaconess Medical Center, Boston, Massachusetts; c Peripheral Interventions, Boston Scientific Corporation, Marlborough, Massachusetts; d Division of Cardiology, Department of Medicine, Beth Israel Deaconess Medical Center, Harvard Medical School, Boston, Massachusetts; e Division of Interventional Radiology, Department of Radiology, Beth Israel Deaconess Medical Center, Harvard Medical School, Boston, Massachusetts

**Keywords:** catheter-based intervention, catheter-directed thrombolysis, clinical research, mechanical thrombectomy, peripheral interventions

## Abstract

**Background:**

In recent years, there has been increasing utilization of catheter-based interventions (CBI) for the treatment of acute pulmonary embolism (PE). We aimed to investigate the trends and practice patterns associated with the utilization of CBI among older patients hospitalized with a PE in US hospitals.

**Methods:**

All Medicare fee-for-service beneficiaries hospitalized with a PE from June 1, 2018, to September 30, 2023, were identified. For outcomes analysis, a subset of patients hospitalized between June 1, 2018, and December 31, 2021, was identified to allow for follow-up to occur.

**Results:**

In total, 313,522 patients were hospitalized with a concomitant PE; of which, 9.12% (n = 28,597) underwent a CBI (4.76% [n = 14,914] catheter-directed thrombolysis and 4.76% [n = 14,918] mechanical thrombectomy). Utilization of CBI increased over time from 5.92% in 2018 to 14.1% in 2023, attributed to increases in mechanical thrombectomy. CBI was used more frequently at teaching hospitals (adjusted OR, 1.10; 95% CI, 1.04-1.15) and in male patients (adjusted OR, 1.15; 95% CI, 1.10-1.21), while less frequently at smaller institutions (adjusted OR, 0.14; 95% CI, 0.09-0.22) and in patients with Black race (adjusted OR, 0.90; 95% CI, 0.85-0.95) or dual enrollment (adjusted OR, 0.80; 95% CI, 0.76-0.86). Older age, Black race, geographic region, and distressed communities were associated with higher rates of death or periprocedural complications.

**Conclusions:**

In this contemporary nationwide analysis of hospitalized patients with PE, we found that rates of CBI increased over time, although uptake of CBI remains heterogeneous. Our findings suggest that there are differences in access to advanced therapies and outcomes among particular populations following CBI for PE management.

## Introduction

Acute pulmonary embolism (PE) is the third leading cause of cardiovascular-related mortality behind acute coronary syndromes and stroke and the rate of PE-related mortality appears to be increasing.^1^^,^^2^ Among the highest risk patients, including those with either right ventricular (RV) dysfunction or hemodynamic instability, the mainstay of treatment continues to be therapeutic anticoagulation with selected patients receiving systemic thrombolysis.^3^^,^^4^ Nevertheless, previous studies have reported rates of major bleeding as high as 10% for systemic thrombolysis, thereby limiting its utilization in practice.^5^ Over the last decade, there has been a significant advance in innovations designed for the interventional management of intermediate and high-risk PE, in particular, catheter-based interventions (CBI).^6^, ^7^, ^8^ CBI for the treatment of PE has the potential to be highly effective within this population, offering rapid improvement in symptoms and hemodynamics with lower risks compared with systemic thrombolysis. However, randomized controlled trials evaluating clinical outcomes such as mortality are still ongoing.^9^^,^^10^

In recent years, numerous CBI modalities for the treatment of PE have emerged US Food and Drug Administration approval. Two systems are currently in practice: catheter-directed thrombolysis (CDT), which involves local administration of thrombolytics directly into the thrombus; and mechanical or aspiration embolectomy, where negative pressure aspiration devices are used to remove the thrombus. Several early stage studies for both categories have been completed and collectively demonstrated that CBI is efficacious at reducing the ratio of the RV and left ventricular (LV) sizes (RV/LV ratio) among patients with intermediate-risk or high-risk PE with acceptable risks of procedural complications.^11^, ^12^, ^13^ Clinical adoption of these devices has been brisk despite the limited available evidence, and a growing number of hospitals across the country are using these devices. Nonetheless, little is known regarding the national patterns of utilization and outcomes associated with CBI in real-world clinical practice beyond the limited clinical trial populations. Additionally, as contemporary practice patterns remain ill-defined in this population, it continues to be unclear whether any health inequities exist with the availability of these devices. As real-world utilization of interventional PE care continues to increase out of proportion to the available evidence, understanding modern clinical practice of this high-risk population has become critical.

In this analysis, we aimed to use comprehensive Medicare claims data to systematically examine the contemporary temporal trends and practice patterns associated with the utilization of CBI among older patients hospitalized for acute PE. In particular, we sought to investigate patient-level and hospital-level factors associated with the utilization of CBI in the Medicare population and, importantly, assess whether any differences in outcomes exist among particular patient populations who undergo these procedures in US hospitals.

## Materials and methods

### Data source and study population

This study was approved by the institutional review board at Beth Israel Deaconess Medical Center (Boston, Massachusetts). Data access was obtained in accordance with the data use agreement between the Center for Medicare and Medicaid Services (CMS) and Beth Israel Deaconess Medical Center.

In this retrospective cohort study, all Medicare fee-for-service (FFS) beneficiaries hospitalized with a concomitant acute PE between June 1, 2018, and September 30, 2023, and those aged >18 years were included in the study cohort. The study initiation date was selected to specifically evaluate the period following the initial approval of novel mechanical thrombectomy devices in the United States. To examine temporal trends and practice variation, patients were identified using the CMS Virtual Research Data Center to query the FFS inpatient data set using International Classification of Diseases, Tenth Revision (ICD-10), Clinical Modification, codes to find hospitalizations with acute PE (I26-) as the first listed diagnosis code. Patients were excluded if they had a nonthrombotic PE such as an air or septic embolism. To avoid counting procedures for alternative acute thrombotic indications rather than PE intervention, patients were additionally excluded if their index admissions also had diagnostic codes attributable to ST-elevation myocardial infarction (STEMI), acute ischemic stroke, or acute limb ischemia. To examine predictors and outcomes, patients were identified by querying the Medicare Provider Analysis and Review file for hospitalizations over the lesser period from June 1, 2018, to December 31, 2021, to allow time for follow-up to occur. Similar inclusion and exclusion criteria were applied with the added exclusion of patients who did not have 1 or more years of enrollment before the index hospitalization or if subgroup information was unavailable. Supplemental Table S1 contains the complete list of billing codes used for this study.

### Patient, procedural, and hospital characteristics

Patient sociodemographic data were collected at the time of the index hospitalization for acute PE using the Master Beneficiary Summary File. Patient baseline comorbidities were ascertained using the CMS Chronic Conditions Warehouse. The remaining comorbidities and baseline characteristics, including markers of disease severity, were determined by historical ICD-10 diagnosis codes reported in Supplemental Table S1. The hospital features reported, including teaching versus nonteaching hospital status, geographic region, and bed capacity were determined via data linkage with the 2020 AHA Annual Survey File.

All procedures were ascertained by ICD-10 Procedure Coding System codes (Supplemental Table S1). The cohort was stratified based on receipt of a catheter-based procedural intervention, systemic thrombolysis, or neither intervention. CBI was further differentiated by catheter modality: CDT or mechanical thrombectomy. Patients with procedural codes for both CBI and systemic thrombolysis were categorized as CBI.

### Outcomes and subgroups

Outcomes analysis was performed among prespecified subgroups. Subgroups included the following: (1) age (stratified in decades), (2) sex, (3) race, (4) geographic region (Northeast, Midwest, South, and West), (5) teaching hospital status (vs nonteaching institutions), (6) rural or urban location, (7) distressed or nondistressed community, and (8) dual enrollment or Medicare alone. Distressed communities were defined through zip code linkage with the distressed community index and defined as those in the 75th percentile or greater on the distressed community index metric.^14^ Race was ascertained based upon patient self-reporting using categories outlined by CMS at the time of enrollment. The outcomes investigated in this study included in-hospital mortality and all-cause mortality assessed at both 30 days and 1 year following the index hospitalization. For patients who underwent CBI, a composite end point of periprocedural safety events was also assessed to evaluate for procedure-related complications. This variable was defined as the composite end point of the receipt of a blood transfusion, gastrointestinal (GI) endoscopic procedure, postprocedural surgical thrombectomy, extracorporeal membrane oxygenation cannulation, mechanical intubation, or presence of an intracranial, GI, or other bleeding event.

### Statistical analysis

Categorical variables are presented as count (percentage) and continuous variables as mean ± SD. To investigate temporal trends, quarterly percentages of CBI utilization were calculated and plotted over the study duration. To investigate the variability in the utilization of catheter-based therapy between hospitals, histogram plots were used to assess the proportion of PE admission for which CBI was used. Representative descriptive statistics were used to assess these institutional patterns including mean, median, and IQR.

Multivariable logistic regression models were applied to investigate patient and hospital characteristics associated with the utilization of CBI and each individual modality. Variables incorporated into the final regression model included patient sociodemographics, chronic comorbidities, markers of disease severity, and hospital characteristics.

Survival methods were used for outcomes analysis. For each subgroup, the cumulative incidence of each end point was calculated, and subgroups were compared with each other via adjusted hazard ratios. In order to derive the time to event variables, patients who did not experience the event of interest were censored at the time of analysis, the last day of FFS coverage, or last day of data availability, whichever came first. All-cause mortality was assessed during the hospital stay, at 30 days, and at 1 year following the index procedures. For these end points, cumulative incidences were estimated using Kaplan-Meier estimates, and hazard ratios were estimated with Cox proportional hazards regression models. Each model was adjusted using inverse probability treatment weighting accounting for patient sociodemographics, chronic comorbidities, markers of disease severity, and hospital characteristics. To analyze 30-day periprocedural safety events, cumulative incidence functions were calculated using Gray subdistributional method to account for the competing risk of death and cause-specific hazard ratios were reported. Similarly, each model was adjusted using inverse probability treatment weighting methods accounting for patient sociodemographics, chronic comorbidities, markers of disease severity, and hospital characteristics.

All analysis were performed using SAS version 9.4 (SAS Institute). A *P* value <.05 was considered statistically significant.

## Results

### Study population selection

Between June 1, 2018, and September 30, 2023, 317,023 Medicare FFS beneficiaries were hospitalized with a billing code for acute PE. Of the identified patients, 64 patients (0.02%) were excluded for a nonthrombotic PE and 3437 patients (1.08%) for a possible alternative indication for thrombolysis (ie, STEMI, acute limb ischemia, or acute ischemic stroke). Following exclusions, 313,522 patients were included in the primary trends analysis cohort. For the outcomes cohort, after exclusions, 199,100 patients were identified between June 1, 2018, and December 31, 2021, and included for analysis (Supplemental Figure S1).

### Temporal trends in device utilization

The Central Illustration depicts the trends in procedural utilization over the course of the study duration. In total, 9.12% (n = 28,597) underwent a CBI, of which, 4.76% (n = 14,914) underwent CDT and 4.76% (n = 14,918) underwent mechanical thrombectomy. Between Q2 of 2018 to Q3 of 2023, CBI utilization increased from 5.92% to 14.1% of patients, demonstrating an 8.2% overall growth. When stratified by treatment modality, mechanical thrombectomy demonstrated the largest increase in procedural utilization, demonstrating a 10.1% growth over the study duration from 1.21% in 2018 Q2 to 11.3% in 2021 Q4. Mechanical thrombectomy became the most frequently used modality for CBI, overtaking CDT in early 2021. Utilization of CDT demonstrated a small decline and systemic thrombolysis remained constant over the period investigated.

**Central Illustration:**
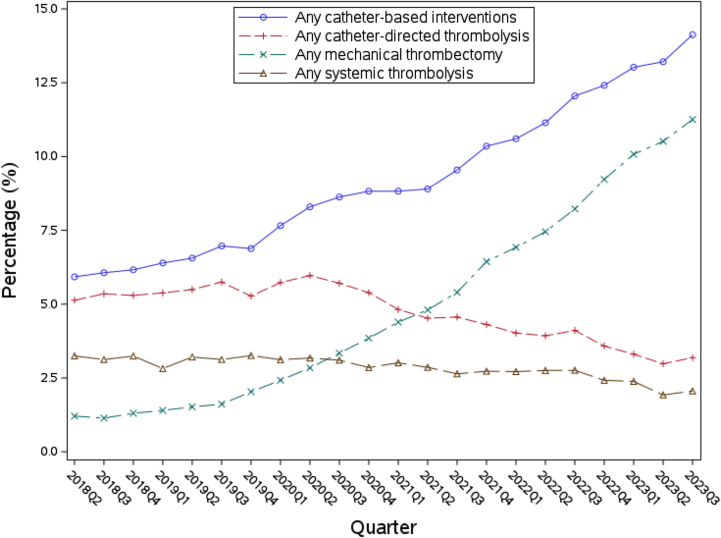
**Temporal trends in the utilization of catheter-based interventions and systemic thrombolysis among hospitalized patients with pulmonary embolism, 2018-2023.**

### Hospital variation in the utilization of catheter-based therapies

**Figure 1:**
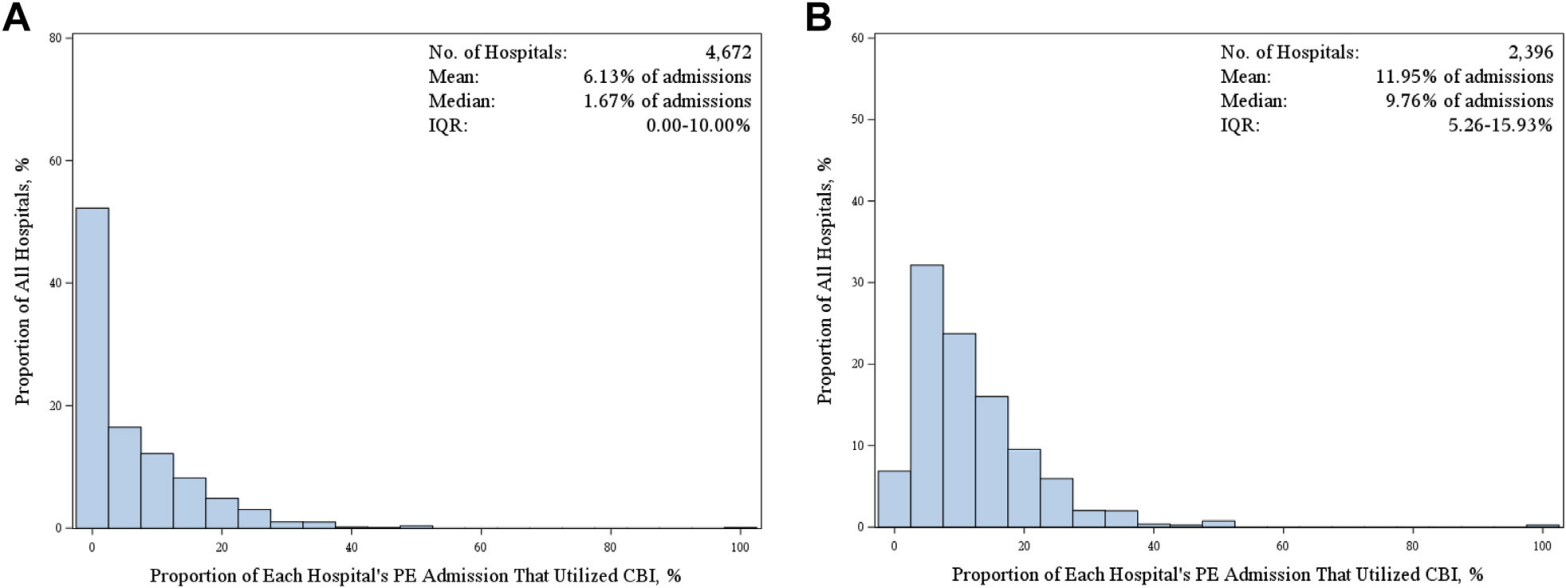
**Hospital variation in the utilization of CBI during PE hospitalizations:** (**A**) all hospitals; (**B**) hospitals which used at least 1 CBI procedure. CBI, catheter-based intervention; PE, pulmonary embolism.

Among 4672 hospitals, 2396 hospitals (51.3%) performed CBI procedures for PE management between 2018 and 2023. Among all studied hospitals, the mean use of CBI was 6.13% of PE admissions (median, 1.67%; IQR, 0%-10.0% of PE admissions). When the analysis was restricted to only hospitals who performed CBI, the median and mean use of CBI was 9.76% (5.26%-15.93%) and 11.9% of PE admissions, respectively (Figure 1). Among individual catheter-based modalities, mechanical thrombectomy was performed numerically at more hospitals as compared with CDT (n = 1748 for mechanical thrombectomy vs n = 1640 for CDT). Among the institutions which performed each procedure, the median (IQR) rates of utilization for CDT and mechanical thrombectomy was 4.27% (1.99%-8.19%) and 4.30% (2.10%-8.33%) of PE admissions, respectively (Supplemental Figures S2 and S3). Over the study duration, the proportion of PE admissions in which systemic thrombolysis was used was less than that of CBI, which included 1906 hospitals with a median (IQR) utilization rate of 3.15% (1.96%-5.00%) of PE admissions (Supplemental Figure S4).

### Baseline characteristics

Table 1 illustrates the baseline characteristics of the study cohort for the outcomes analysis (June 1, 2018, to December 31, 2021) stratified by the primary treatment modality used. Among the study cohort, the mean age was 74.2 ± 11.1 years, 54.6% (n = 108,614) were female, 82.5% (n = 164,208) were of White race, and 16.4% (n = 32,623) were dually enrolled in both Medicare and Medicaid. Patients were managed in a range of hospital settings including 4.1% (n = 8176) in rural location and 73.0% (n = 145,258) in a teaching institution. Cardiovascular comorbidities were frequent in the cohort, including 7.1% (n = 14,043) with a previous myocardial infarction, 15.3% (n = 30,381) with atrial fibrillation, 34.8% (n = 69,284) with congestive heart failure, 39.7% (n = 78,963) with diabetes mellitus, 52.8% (n = 105,209) with ischemic heart disease, 77.4% (n = 154,077) with hyperlipidemia, 82.7% (n = 164,618) with hypertension, and 43.1% (n = 85,726) with obesity. Additionally, 4.7% (n = 9260) of included patients had a history of a previous PE, and 1.9% (n = 3786) had a history of deep vein thrombosis (DVT). A total of 0.2% (n = 472) of patients had a previous intracranial hemorrhage and 0.9% (n = 1867) of patients had a previous GI bleeding event.

**Table 1:** Baseline characteristics of patients with acute pulmonary embolism, stratified by primary treatment modality.

Patient characteristics	Catheter-based intervention (n = 15,144)	Systemic thrombolysis (n = 4214)	Neither intervention (n = 179,742)	All patients (N = 199,100)
Age, y	71.9 ± 9.4	71.4 ± 11.2	74.5 ± 11.2	74.2 ± 11.1
Female sex	7631 (50.4)	2303 (54.7)	98,680 (54.9)	108,614 (54.6)
Race/ethnicity				
White	12,503 (82.6)	3242 (76.9)	148,463 (82.6)	164,208 (82.5)
Black	1996 (13.2)	756 (17.9)	23,259 (12.9)	26,011 (13.1)
Asian	55 (0.4)	25 (0.6)	1194 (0.7)	1274 (0.6)
Other	590 (3.9)	191 (4.5)	6826 (3.8)	7607 (3.8)
Dual enrollment status	1896 (12.5)	752 (17.8)	29,975 (16.7)	32,623 (16.4)
Distressed community	3036 (20.0)	869 (20.6)	36,362 (20.2)	40,267 (20.2)
Rural location	617 (4.1)	119 (2.8)	7440 (4.1)	8176 (4.1)
Geographic region				
Northeast	1892 (12.5)	846 (20.1)	32,784 (18.2)	35,522 (17.8)
Midwest	2737 (18.1)	505 (12.0)	26,324 (14.6)	29,566 (14.8)
South	8249 (54.5)	2053 (48.7)	89,536 (49.8)	99,838 (50.1)
West	2266 (15.0)	810 (19.2)	31,098 (17.3)	34,174 (17.2)
Comorbidities				
Acute myocardial infarction	585 (3.9)	223 (5.3)	13,235 (7.4)	14,043 (7.1)
Anemia	7108 (46.9)	2251 (53.4)	113,074 (62.9)	122,433 (61.5)
Asthma	2234 (14.8)	707 (16.8)	36,113 (20.1)	39,054 (19.6)
Atrial fibrillation	1268 (8.4)	438 (10.4)	28,675 (16.0)	30,381 (15.3)
Breast cancer	903 (6.0)	250 (5.9)	13,662 (7.6)	14,815 (7.4)
Colorectal cancer	442 (2.9)	127 (3.0)	7795 (4.3)	8364 (4.2)
Endometrial cancer	250 (1.7)	67 (1.6)	3346 (1.9)	3663 (1.8)
Lung cancer	387 (2.6)	139 (3.3)	11,384 (6.3)	11,910 (6.0)
Prostate cancer	985 (6.5)	221 (5.2)	12,522 (7.0)	13,728 (6.9)
Chronic kidney disease	5827 (38.5)	1854 (44.0)	86,339 (48.0)	94,020 (47.2)
Congestive heart failure	3421 (22.6)	1227 (29.1)	64,636 (36.0)	69,284 (34.8)
COPD/bronchiectasis	3473 (22.9)	1147 (27.2)	66,886 (37.2)	71,506 (35.9)
Diabetes	5538 (36.6)	1756 (41.7)	71,669 (39.9)	78,963 (39.7)
Hip/pelvic fracture	426 (2.8)	182 (4.3)	10,231 (5.7)	10,839 (5.4)
Hyperlipidemia	10,939 (72.2)	2994 (71.0)	140,144 (78.0)	154,077 (77.4)
Hypertension	11,731 (77.5)	3294 (78.2)	149,593 (83.2)	164,618 (82.7)
Ischemic heart disease	6086 (40.2)	1836 (43.6)	97,287 (54.1)	105,209 (52.8)
Leukemia and lymphomas	379 (2.5)	131 (3.1)	7100 (4.0)	7610 (3.8)
Liver disease, cirrhosis, or other liver condition	1738 (11.5)	578 (13.7)	29,295 (16.3)	31,611 (15.9)
Obesity	7403 (48.9)	1918 (45.5)	76,405 (42.5)	85,726 (43.1)
Osteoporosis	2259 (14.9)	691 (16.4)	39,522 (22.0)	42,472 (21.3)
Peripheral vascular disease	2868 (18.9)	1012 (24.0)	51,029 (28.4)	54,909 (27.6)
Stroke/TIA	1732 (11.4)	566 (13.4)	33,042 (18.4)	35,340 (17.7)
Tobacco use disorders	2098 (13.9)	669 (15.9)	36,006 (20.0)	38,773 (19.5)
Previous PE	607 (4.0)	181 (4.3)	8472 (4.7)	9260 (4.7)
Previous DVT	275 (1.8)	82 (1.9)	3429 (1.9)	3786 (1.9)
History of intracranial bleed	20 (0.1)	6 (0.1)	446 (0.2)	472 (0.2)
History of GI bleed	77 (0.5)	23 (0.5)	1767 (1.0)	1867 (0.9)
Markers of disease severity				
Acute cor pulmonale	6277 (41.4)	1219 (28.9)	12,696 (7.1)	20,192 (10.1)
Hypotension	1533 (10.1)	642 (15.2)	9448 (5.3)	11,623 (5.8)
Shock	657 (4.3)	741 (17.6)	1905 (1.1)	3303 (1.7)
Cardiac arrest	505 (3.3)	794 (18.8)	2363 (1.3)	3662 (1.8)
Need for ECMO	66 (0.4)	21 (0.5)	71 (0.0)	158 (0.1)
Need for mechanical ventilation	972 (6.4)	1166 (27.7)	4334 (2.4)	6472 (3.3)
Need for vasopressors	377 (2.5)	507 (12.0)	1348 (0.7)	2232 (1.1)
Teaching hospital	12,184 (80.5)	3320 (78.8)	129,754 (72.2)	145,258 (73.0)
Bed size				
6-24	21 (0.1)	4 (0.1)	1890 (1.1)	1915 (1.0)
25-49	124 (0.8)	43 (1.0)	6818 (3.8)	6985 (3.5)
50-99	446 (2.9)	165 (3.9)	11,811 (6.6)	12,422 (6.2)
100-199	2206 (14.6)	666 (15.8)	34,506 (19.2)	37,378 (18.8)
200-299	2847 (18.8)	769 (18.2)	33,604 (18.7)	37,220 (18.7)
300-399	2654 (17.5)	678 (16.1)	25,898 (14.4)	29,230 (14.7)
400-499	1940 (12.8)	397 (9.4)	17,122 (9.5)	19,459 (9.8)
≥500	4906 (32.4)	1492 (35.4)	48,093 (26.8)	54,491 (27.4)

Values are mean ± SD or n (%).

COPD, chronic obstructive pulmonary disease; DVT, deep vein thrombosis; ECMO, extracorporeal membrane oxygenation; GI, gastrointestinal; PE, pulmonary embolism; TIA, transient ischemic attack.

### Patient, hospital, and geographic factors associated with device utilization

**Figure 2:**
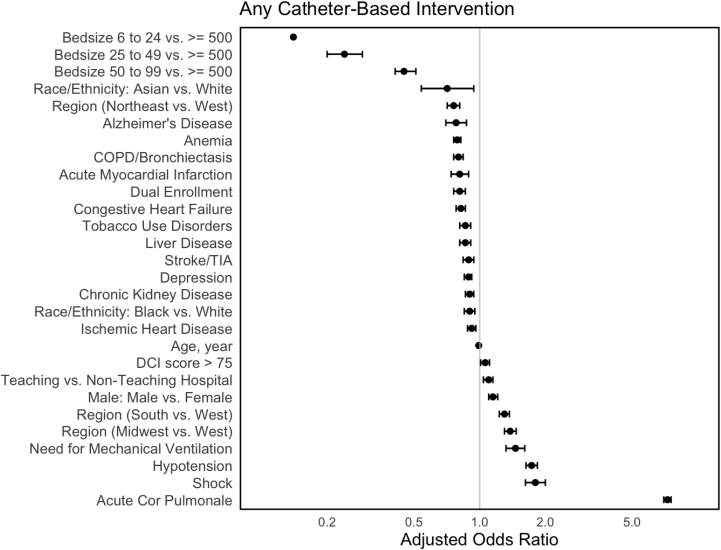
**Selected characteristics associated with the utilization of any catheter-based intervention.** DCI, distressed community index; TIA, transient ischemic attack.

**Figure 3:**
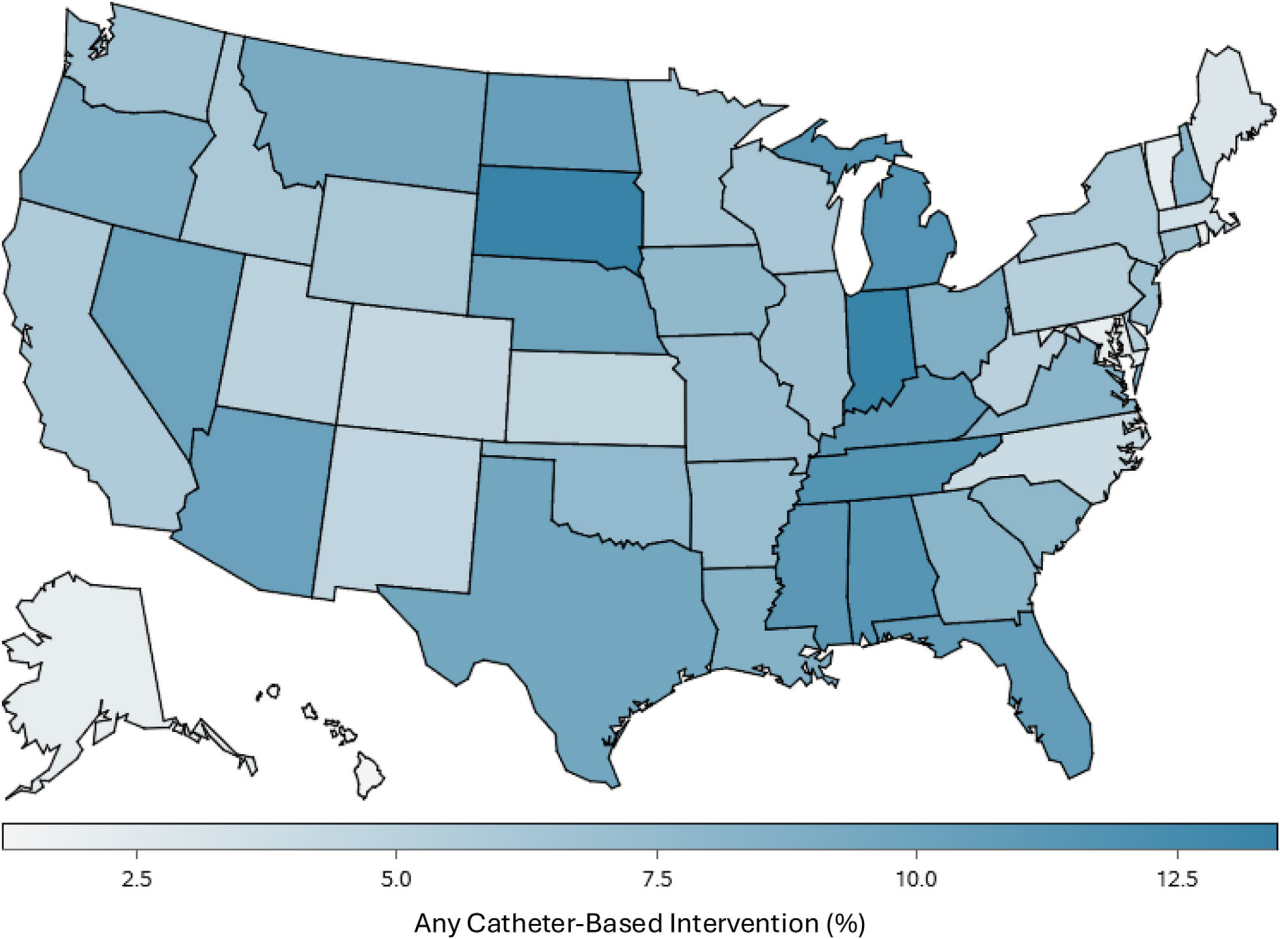
**Heat map demonstrating the relative percentage of pulmonary embolism admissions where a catheter-based intervention was used**.

Figure 2 depicts the results of the multivariable model identifying predictors of CBI utilization. Following multivariable adjustment, the factors most strongly associated with the utilization of CBI included patient presentation with acute cor pulmonale (adjusted OR, 7.26; 95% CI, 6.98-7.54; *P* < .01), shock (adjusted OR, 1.80; 95% CI, 1.62-2.00; *P* < .01), hypotension (adjusted OR, 1.73; 95% CI, 1.63-1.84; *P* < .01), need for mechanical ventilation (adjusted OR, 1.46; 95% CI, 1.32-1.61; *P* < .01), male sex (adjusted OR, 1.15; 95% CI, 1.10-1.21; *P* < .01), and patients treated at teaching institutions (adjusted OR, 1.10; 95% CI, 1.04-1.15; *P* < .01). Smaller hospitals by bed size were least associated with the utilization of CBI (6-24 vs 500+ beds; adjusted OR, 0.14; 95% CI, 0.09-0.22; *P* < .01). Additionally, patients who identified as Black race (adjusted OR, 0.90; 95% CI, 0.85-0.95; *P* < .01), dually enrolled on Medicare and Medicaid (adjusted OR, 0.80; 95% CI, 0.76-0.86; *P* < .01), and patients who were younger (adjusted OR, 0.99; 95% CI, 0.98 - 0.99; *P* < .01) were less likely to receive CBI. Moreover, CBI was used more frequently in Southern and Midwestern US states, with the highest rates of utilization in Indiana, Tennessee, Missouri, and Alabama. CBI was least frequently used among Northeastern states including Massachusetts and Maryland (Figure 3).

Supplemental Figure S5 depicts the patient and hospital features associated with the utilization of individual CBI modalities including CDT and mechanical thrombectomy. Utilization of both modalities were strongly associated with markers of disease severity including acute cor pulmonale, hypotension and shock. History of an intracranial bleed was negatively associated with the receipt of CDT therapy (adjusted OR, 0.23; 95% CI, 0.09-0.63; *P* < .01) and trended toward a positive association with the utilization of mechanical thrombectomy (adjusted OR, 1.53; 95% CI, 0.91-2.57; *P* = .11).

### Outcomes and subgroup analysis

Among patients who underwent CBI, in-hospital mortality occurred in 4.8% (95% CI, 4.5%-5.2%), 30-day mortality in 6.8% (95% CI, 7.0%-7.8%), and 1-year mortality in 14.1% (95% CI, 13.5%-14.6%). Following CBI, the rate of 30-day periprocedural complications was 14.9% (95% CI, 14.3%-15.4%). In comparison, among patients who underwent systemic thrombolysis, in-hospital mortality was 21.1% (95% CI, 19.8%-22.3%), 30-day mortality was 25.2% (95% CI, 23.8%-26.5%), and 30-day complications was 21.9% (95% CI, 20.6%-23.2%). Among patients who underwent CDT and mechanical thrombectomy, 30-day mortality was 6.2% (95% CI, 5.8%-6.7%) and 8.8% (95% CI, 8.0%-9.5%), respectively.

Supplemental Tables S2 to S8 illustrate the results of the subgroup analysis. Among patients who underwent CBI, older age (85 years or older vs 65 years and younger) was associated with higher rates of in-hospital mortality (5.9% vs 2.6%; adjusted HR, 2.50; 95% CI, 2.19-2.57), 30-day mortality (11.9% vs 3.9%; adjusted HR, 3.18; 95% CI, 2.44-4.15), 1-year mortality (28.7% vs 14.5%; adjusted HR, 2.23; 95% CI, 1.91-2.60), and 30-day periprocedural complications (12.1% vs 8.5%; cause-specific adjusted HR, 1.47; 95% CI, 1.20-1.80) (Supplemental Table S2). Following CBI, Black patients (relative to White patients) had higher rates of death at 1 year (14.9% vs 17.6%; adjusted HR, 1.16; 95% CI, 1.03-1.29) (Supplemental Table S4). Additionally, after CBI procedures, patients from a distressed community (10.4% vs 9.07%; cause-specific adjusted HR, 1.14; 95% CI, 1.01-1.30) and southern (vs northeastern) states (10.2% vs 8.7%; cause-specific adjusted HR, 1.18; 95% CI, 1.02-1.37) had higher rates of 30-day periprocedural complications (Supplemental Tables S5 and S7). Patients managed at teaching institutions had higher rates of in-hospital mortality (4.2% vs 2.8%; adjusted HR, 1.53; 95% CI, 1.23-1.89) but lower rates of 30-day periprocedural complications (9.60% vs 10.8%, cause-specific adjusted HR, 0.89; 95% CI, 0.79-0.99).

## Discussion

In this large, nationwide study of Medicare beneficiaries, we aimed to investigate the temporal trends and practice patterns associated with the utilization of CBI for acute PE in US hospitals. Our findings indicate that while CBI continues to be used in only a minority of PE hospitalizations, overall rates increased over the study duration, primarily driven by the growing adoption of mechanical thrombectomy. Nevertheless, our analysis demonstrates that significant interhospital variation exists in the uptake of CBI across the United States, with the highest utilization at larger institutions, teaching hospitals, and for patients with high-risk features. Moreover, our findings also indicate that differences exist in access to CBI including lower rates of CBI utilization in those who identified as Black race, had dual enrollment, or were of female sex. Lastly, we found several inequities in outcomes following CBI procedures, most notably among older patients, Black patients, and those from distressed communities and particular geographic regions.

Over the past decade, CBI has become an increasingly attractive modality for the management of intermediate-high and high-risk PE.^15^^,^^16^ In a randomized controlled trial comparing ultrasound-assisted CDT with anticoagulation alone for intermediate-risk patients, CDT was superior to anticoagulation alone in reducing the RV/LV ratio within 24 hours.^13^ Similar findings have also been demonstrated for catheter-based mechanical thrombectomy approaches in registries, including the recently completed FLAME study, which demonstrated lower in-hospital mortality for patients with high-risk PE managed with mechanical thrombectomy compared with a historical control.^9^^,^^11^ While encouraging, these data are limited by small sample sizes and nonmortality end points making investigations of real-world utilization essential. Additionally, with the emergence of artificial intelligence approaches to more rapidly identify right heart strain and high-risk patients, the candidate pool of patients for may CBI may be increasing. In the present analysis, we investigated more than 300,000 PE hospitalizations, of which nearly 30,000 CBI procedures were performed. Our findings indicate that CBI utilization continues to grow among Medicare beneficiaries. Most notably, by 2021, mechanical thrombectomy became the most frequently utilized advanced therapeutic modality in our cohort in less than 3 years after approval. However, this was likely influenced by the older patient population studied in this analysis. Given the intrinsically higher bleeding risk of this group, it is plausible that mechanical thrombectomy was utilized more frequently than CDT and systemic thrombolysis to mitigate this risk.^17^^,^^18^ This finding is particularly timely as there is growing evidence that large-bore mechanical thrombectomy may be associated with lower rates of clinical deterioration and ICU utilization among patients with intermediate-risk PE based on the recently reported out PEERLESS trial.^19^ Of note, mechanical thrombectomy is also being investigated for application in other VTE conditions beyond PE including iliofemoral DVT in the ongoing DEFIANCE clinical trial.^20^ Importantly, our findings indicate that CBI offered advanced therapeutic management to a growing number of patients that otherwise would have had limited options beyond anticoagulation. Nonetheless, it remains incompletely understood how outcomes following CDT, mechanical thrombectomy, and systemic thrombolysis are compared with each other as randomized data are not yet available.

There was notable interhospital variability in the use of CBI as observed in the more than 4500 hospitals studied in this analysis. When specifically examining which variables were associated with utilization of CBI, we found that catheter interventions were more commonly used at larger, teaching institutions and in Southern and Midwestern states, which mimics patterns consistent with prior work of endovascular-based cardiovascular devices.^21^^,^^22^ These findings are also worth commenting on, as it has been previously shown that low-volume centers have higher rates of in-hospital mortality following PE intervention.^23^ These findings may be partially explained by the lack of cohesive guidelines around interventional PE procedures. Furthermore, we also investigated clinical variables associated with each interventional modality. While both CDT and mechanical thrombectomy shared many of the same predictors, a history of intracranial bleeding was a strong negative predictor for the use of CDT. These data suggest that in real-world clinical practice, operators are not necessarily utilizing CDT in patients with the highest bleeding risk, which may explain the comparatively low rates of major bleeding that have been associated with this modality in recent observational studies.^24^ Nevertheless, this hypothesis requires further investigation.

Moreover, prior to this analysis, limited data existed regarding how access and outcomes in CBI care vary for different patient populations with PE in the United States. For example, in 1 study that leveraged data from the National Inpatient Sample to investigate patients who underwent CDT, all races had comparable rates of in-hospital mortality and major bleeding.^25^ These findings were also evident in another study where the investigators did not find the existence of health inequities, including sex, household income, insurance status, or geographic region, to be associated with mortality following catheter-based or systemic thrombolysis.^26^ In the present study, we contrast these previous observations in multiple ways. First, we found that at a national scale, older patients who identified as Black race, had dual enrollment, or were of female sex were significantly less likely to receive CBI. Importantly, we also observed differences in outcomes following CBI among numerous disadvantaged groups including those with older age, Black race, and those residing in particular geographic regions and distressed communities. While many of these characteristics have been implicated as disparities in the management of other cardiac conditions including structural, peripheral, and coronary heart disease, further analysis into these gaps in access to and outcomes following CBI are necessary.^27^, ^28^, ^29^, ^30^

Our findings must be interpreted within the context of several limitations. First, this study contains inherent limitations, which are present in all nationwide, claims-based analyses. Namely, as patients were identified utilizing insurance billing codes, we were limited in our ability to fully adjudicate diagnoses, and there remains the possibility of misclassification or underreporting of claims codes. Recent work has exemplified the limitations of utilizing ICD codes for identifying PE.^31^ Second, while this analysis was able to capture a nationwide perspective, we were able to study only patients enrolled in Medicare, which resulted in a relatively older population. As such, this study underestimates the total CBI volume in the United States, and the findings may not apply to a younger, non–Medicare-insured population. Third, we did not have access to granular procedural details such as the brand of the catheter utilized for the PE intervention or detailed information regarding the PE disease burden (ie, degree of RV dysfunction or location of the embolus). As a result, we were limited in our ability to calculate each patient’s individual risk status using contemporary risk stratification as imaging and cardiac biomarkers were not available. Lastly, while we excluded patients who may have had an alternative procedural indication (ie, acute limb ischemia, STEMI, and ischemic stroke), the possibility of misclassification for alternative indications rather than PE intervention remains a possibility.

## Conclusion

In this broad, contemporary analysis of CBI therapy for PE management, we highlight the current landscape of interventional PE therapy for older patients in the United States. First, we observed an overall increase in CBI utilization over the study duration between 2018 and 2023, most notably for mechanical thrombectomy platforms. These devices became the most utilized CBI modality in the Medicare population by the end of the study period. Additionally, we found that use of CBI varied substantially across United States hospitals and occurred more frequently at larger institutions, teaching hospitals, and for higher-risk patients. We also observed access to CBI therapy varied across different patient populations including lower rates of CBI utilization in those who identified as Black race, had dual enrollment, or were of female sex. Finally, we found that differences existed in outcomes following CBI, such as worse outcomes among older patients, Black patients, and those who lived in distressed communities and particular geographic regions. Future studies to externally reproduce these patterns of utilization and elucidate the reasons for the observed variations in outcomes are needed.
