# Supplementary material for: Temporal Trends and Practice Patterns Associated With Utilization of Catheter-Based Interventions for Pulmonary Embolism
Source: J Soc Cardiovasc Angiogr Interv. 2025 Jul 23;4(8):103736. doi: 10.1016/j.jscai.2025.103736 (PMC12462096; doi:10.1016/j.jscai.2025.103736)
Supplement: Supplementary Material [file mmc1.docx]

**Supplemental Information:**

**Temporal Trends and Practice Patterns Associated with the Utilization of Catheter-Based Interventions for Pulmonary Embolism**

Nathan W. Watson, MD^1,2^, Michael R. Jaff, DO^3^, Brett J. Carroll, MD^2,4^, Hibiki Orui, MA^2^, Siling Li, MSc^2^, Yang Song, MSc^2^, Jeffrey L. Weinstein, MD^5^, Robert W. Yeh, MD, MSc^2,4^, Eric A. Secemsky, MD, MSc^2,4^

**Affiliations:**

^1^ Department of Medicine, Brigham and Women’s Hospital, Harvard Medical School, Boston, MA

^2^ Richard A. and Susan F. Smith Center for Outcomes Research in Cardiology, Department of Medicine, Beth Israel Deaconess Medical Center, Boston, MA

^3^ Boston Scientific Corporation, Marlborough, MA

^4^ Division of Cardiology, Department of Medicine, Beth Israel Deaconess Medical Center, Harvard Medical School, Boston, MA

^5^ Division of Interventional Radiology, Department of Radiology, Beth Israel Deaconess Medical Center, Harvard Medical School, Boston, MA

**SUPPLEMENTAL TABLES**

**Supplemental Table 1:** Diagnostic and procedure billing codes utilized for analysis of CBI among Medicare beneficiaries.

| **Diagnosis/ Procedure** | **ICD-10 CM/ PCS Code** |
| --- | --- |
| Acute pulmonary embolism | I26 (excluding I26.01, I26.90) |
| **Catheter-Based Interventions and Systemic Thrombolysis** |  |
| Systemic Thrombolysis | 3E04017: Introduction of Other Thrombolytic into Central Vein, Open Approach  3E04317: Introduction of Other Thrombolytic into Central Vein, Percutaneous Approach  3E03017: Introduction of Other Thrombolytic into Peripheral Vein, Open Approach  3E03317: Introduction of Other Thrombolytic into Peripheral Vein, Percutaneous Approach |
| Mechanical Aspiration Thrombectomy | 02CP3ZZ: Extirpation of Matter from Pulmonary Trunk, Percutaneous Approach  02CQ3ZZ: Extirpation of Matter from Right Pulmonary Artery, Percutaneous Approach  02CR3ZZ: Extirpation of Matter from Left Pulmonary Artery, Percutaneous Approach  02CQ4ZZ: Extirpation of Matter from Right Pulmonary Artery, Percutaneous Endoscopic Approach  02CR4ZZ: Extirpation of Matter from Left Pulmonary Artery, Percutaneous Endoscopic Approach  02CP4ZZ: Extirpation of Matter from Pulmonary Trunk, Percutaneous Endoscopic Approach |
| Catheter-Directed Thrombolysis | 3E06017: Introduction of Other Thrombolytic into Central Artery, Open Approach  3E06317: Introduction of Other Thrombolytic into Central Artery, Percutaneous Approach  02FP3ZZ: Fragmentation of Pulmonary Trunk, Percutaneous Approach  02FP3Z0: Fragmentation of Pulmonary Trunk, Percutaneous Approach, Ultrasonic  02FQ3ZZ: Fragmentation of Right Pulmonary Artery, Percutaneous Approach  02FQ3Z0: Fragmentation of Right Pulmonary Artery, Percutaneous Approach, Ultrasonic  02FR3ZZ: Fragmentation of Left Pulmonary Artery, Percutaneous Approach  02FR3Z0: Fragmentation of Left Pulmonary Artery, Percutaneous Approach, Ultrasonic |
| Ultrasound-Assisted CDT | 6A75, 6A750, 6A750Z, 6A751, 6A751Z, 6A750Z6, 6A751Z6, 6A750Z7, 6A751Z7, 6A750ZZ, 6A751ZZ |
| **PE Severity** |  |
| **Acute cor pulmonale** | I26.02, I26.09 |
| **Hypotension** |  |
| Other hypotension | I95.89 |
| Hypotension, unspecified | I95.9 |
| **Shock** |  |
| Cardiogenic shock | R57.0 |
| Other or unspecified shock | R57.8, R57.9 |
| **Cardiac Arrest** | I46 (I46.0 – I46.9) |
| Need for ECMO | 5A1522F, 5A15A2F, 5A1522G, 5A1522H, 5A15A2G, 5A15A2H |
| **Need for Mechanical Ventilation** | 5A1935Z, 5A1945Z, 5A1955Z |
| **Vasopressors utilized** | 3E030XZ, 3E033XZ, 3E040XZ, 3E043XZ, 3E050XZ, 3E053XZ, 3E060XZ, 3E063XZ |
| **Adverse Events** |  |
| **GI Bleed** |  |
| Esophageal varices with bleeding | I85.01, I85.11 |
| Other diseases of esophagus with bleeding | K22.11 |
| Acute gastric ulcer with bleeding | K25.0, K25.2 |
| Chronic gastric ulcer with bleeding | K25.4, K25.6 |
| Acute duodenal ulcer with bleeding | K26.0, K26.2 |
| Chronic duodenal ulcer with bleeding | K26.4, K26.6 |
| Acute peptic ulcer with bleeding | K27.0, K27.2 |
| Chronic peptic ulcer with bleeding | K27.4, K27.6 |
| Acute duodenal ulcer with bleeding | K28.0, K28.2 |
| Chronic duodenal ulcer with bleeding | K28.4, K28.6 |
| Gastritis or duodenitis with bleeding | K29.01, K29.21, K29.31, K29.41, K29.51, K29.61, K29.71, K29.81, K29.91 |
| Angiodysplasia of stomach and duodenum with bleeding | K31.811 |
| Hematemesis | K92.0 |
| Gastrointestinal hemorrhage, unspecified | K92.2 |
| **Intracranial Hemorrhage** |  |
| Nontraumatic subarachnoid hemorrhage | I60 (I60.0 – I60.9) |
| Nontraumatic intracerebral hemorrhage | I61 (I61.0 – I61.9) |
| Other and unspecified nontraumatic intracranial hemorrhage | I62 (I62.0 – I62.9) |
| **Other Bleeding** |  |
| Recurrent and persistent hematuria | N02 (N02.0 – N02.9) |
| Hematuria | R31 (R31.0 – R31.9) |
| Congestion and hemorrhage of prostate | N42.1 |
| Abnormal vaginal or uterine bleeding | N93.8, N93.9 |
| Excessive bleeding in the premenopausal period | N92.4 |
| Postmenopausal bleeding | N95.0 |
| Hemorrhage from respiratory passages | R04 (R04.0 – R04.9) |
| Hemorrhage, not elsewhere classified | R58 |
| Retinal hemorrhage | H35.6 (H35.60 – H35.63) |
| Vitreous hemorrhage | H43.1 (H43.10 – H43.13) |
| Hyphema | H21.0 (H21.00 – H24.00) |
| Choroidal hemorrhage and rupture | H31.3 (H31.30 – H31.32) |
| Otorrhagia | H92.2 (H92.20 – H92.23) |
| Hemopericardium | I32.3 |
| Hemothorax | J94.2 |
| Hemarthrosis | M25.0 (M25.00 – M25.08) |
| **Need for Blood Transfusion** |  |
| Whole Blood | 30230H, 30230H0, 30230H1, 30233H, 30233H0, 30233H1, 30240H, 30240H0, 30240H1, 30243H, 30243H0, 30243H1 |
| Red Blood Cells | 30230N, 30230N0, 30230N1, 30233N, 30233N0, 30233N1, 30240N, 30240N0, 30240N1, 30243N, 30243N0, 30243N1 |
| **Need for GI Endoscopy** |  |
| EGD | 0DJ08ZZ, 0DJ68ZZ |
| Colonoscopy | 0DJD8ZZ |
| **Need for Mechanical Ventilation** | 5A1935Z, 5A1945Z, 5A1955Z |
| **Need for Surgical Thrombectomy** | 02CP0ZZ, 02CQ0ZZ, 02CR0ZZ |
| **Need for post-procedural ECMO** | 5A1522F, 5A15A2F, 5A1522G, 5A1522H, 5A15A2G, 5A15A2H |
| **Comorbidities** |  |
| Prior PE | I26 (excluding I26.01, I26.90), Z86.711, I27.24, I27.82 |
| Prior DVT | I82.220, I82.221, I82.401, I82.402, I82.403, I82.409, I82.411, I82.412, I82.413, I82.419, I82.421, I82.422, I82.423, I82.429, I82.431, I82.432, I82.433, I82.439, I82.441, I82.442, I82.443, I82.449, I82.451, I82.452, I82.453, I82.459, I82.461, I82.462, I82.463, I82.469, I82.491, I82.492, I82.493, I82.499, I82.4Y1, I82.4Y2, I82.4Y3, I82.4Y9, I82.4Z1, I82.4Z2, I82.4Z3, I82.4Z9, I82.501, I82.502, I82.503, I82.509, I82.511, I82.512, I82.513, I82.519, I82.521, I82.522, I82.523, I82.529, I82.531, I82.532, I82.533, I82.539, I82.541, I82.542, I82.543, I82.549, I82.551, I82.552, I82.553, I82.559, I82.561, I82.562, I82.563, I82.569, I82.591, I82.592, I82.593, I82.599, I82.5Y1, I82.5Y2, I82.5Y3, I82.5Y9, I82.5Z1, I82.5Z2, I82.5Z3, I82.5Z9 |
| **Readmissions** |  |
| Venous Thromboembolism-Related | I26 (excluding I26.01, I26.90), I27.24, I27.82, I82.220, I82.221, I82.401, I82.402, I82.403, I82.409, I82.411, I82.412, I82.413, I82.419, I82.421, I82.422, I82.423, I82.429, I82.431, I82.432, I82.433, I82.439, I82.441, I82.442, I82.443, I82.449, I82.451, I82.452, I82.453, I82.459, I82.461, I82.462, I82.463, I82.469, I82.491, I82.492, I82.493, I82.499, I82.4Y1, I82.4Y2, I82.4Y3, I82.4Y9, I82.4Z1, I82.4Z2, I82.4Z3, I82.4Z9, I82.501, I82.502, I82.503, I82.509, I82.511, I82.512, I82.513, I82.519, I82.521, I82.522, I82.523, I82.529, I82.531, I82.532, I82.533, I82.539, I82.541, I82.542, I82.543, I82.549, I82.551, I82.552, I82.553, I82.559, I82.561, I82.562, I82.563, I82.569, I82.591, I82.592, I82.593, I82.599, I82.5Y1, I82.5Y2, I82.5Y3, I82.5Y9, I82.5Z1, I82.5Z2, I82.5Z3, I82.5Z9 |
| Pulmonary Hypertension-Related | I27.0, I27.2 |
| Congestive Heart Failure-Related | I11.0, I13.0, I13.2, I50.1, I50.20, I50.21, I50.22, I50.23, I50.30, I50.31, I50.32, I50.33, I50.40, I50.41, I50.42, I50.43, I50.810, I50.811, I50.812, I50.813, I50.814, I50.82, I50.83, I50.84, I50.89, I50.9 |
| **Exclusion Criteria** |  |
| **STEMI** |  |
| ST elevation (STEMI) myocardial infarction of anterior wall | I21.0 (I21.01 – I21.09) |
| ST elevation (STEMI) myocardial infarction of inferior wall | I21.1 (I21.11, I21.19) |
| ST elevation (STEMI) myocardial infarction of other sites | I21.2 (I21.21, I21.29) |
| Subsequent ST elevation (STEMI) and non-ST elevation (NSTEMI) myocardial infarction | I22 (I22.0 – I22.9) |
| **Acute ischemic stroke** |  |
| Cerebral infarction due to thrombosis of precerebral arteries | I63.0 (I63.00 – I63.09) |
| Cerebral infarction due to embolism of precerebral arteries | I63.1 (I63.10 – I63.19) |
| Cerebral infarction due to unspecified occlusion of stenosis of precerebral arteries | I63.2 (I63.20 – I63.29) |
| Cerebral infarction due to thrombosis of cerebral arteries | I63.3 (I63.0 – I63.9) |
| Cerebral infarction due to embolism of cerebral arteries | I63.4 (I63.40 – I63.49) |
| Cerebral infarction due to unspecified occlusion or stenosis of cerebral arteries | I63.5 (I63.50 – I63.59) |
| Cerebral infarction due to cerebral venous thrombosis, nonpyogenic | I63.6 |
| Other cerebral infarction | I63.8 (I63.81, I63.89) |
| Cerebral infarction, unspecified | I63.9 |
| Acute cerebrovascular insufficiency | I67.81 |
| Cerebral ischemia | I67.82 |
| **Acute Limb Ischemia** | I743, I745, I75021, I75022, I75023, I75029 |
| **Air Embolism** | T80.0 |
| **Septic Embolism** | I26.01, I26.90 |

**Supplemental Table 2:** Cumulative incidence and adjusted hazard ratios for patients who underwent catheter-based interventions, stratified by age.

| **Subject Characteristic** | **65 or younger (N = 27,488)** | **66-74 (N = 71,009)** | **75-84 (N = 62,437)** | **85 or older (N = 33,952)** | **Adjusted HR**  **(66-74 vs 65 or younger)** | **Adjusted HR**  **(75-84 vs 65 or younger)** | **Adjusted HR**  **(85 or older vs 65 or younger)** |
| --- | --- | --- | --- | --- | --- | --- | --- |
| **Catheter-Based Intervention** |  |  |  |  |  |  |  |
| In-Hospital Mortality^1^ | 49 (2.6%)  (1.9%-3.4%) | 186 (3.2%)  (2.7%-3.6%) | 223 (4.2%)  (3.7%-4.8%) | 101 (5.9%)  (4.8%-7.1%) | 1.24 (1.15-1.34) | 1.77 (1.56-1.82) | 2.50 (2.19-2.57) |
| 30-day all-cause mortality | 73 (3.9%)  (3.0%-4.7%) | 310 (5.3%)  (4.7%-5.9%) | 368 (7.1%)  (6.4%-7.8%) | 204 (11.9%)  (10.4%-13.5%) | 1.38 (1.08-1.78) | 1.85 (1.44-2.37) | 3.18 (2.44-4.15) |
| One-year all-cause mortality | 239 (14.5%)  (12.7%-16.2%) | 741 (14.1%)  (13.2%-15.1%) | 935 (19.7%)  (18.6%-20.9%) | 443 (28.7%)  (26.4%-31.0%) | 1.01 (0.87-1.16) | 1.45 (1.26-1.67) | 2.23 (1.91-2.60) |
| 30-day perioperative safety event^2^ | 161 (8.5%)  (7.3%-9.8%) | 513 (8.8%)  (8.0%-9.5%) | 575 (11.0%)  (10.1%-11.8%) | 208 (12.1%)  (10.5%-13.6%) | 1.03 (0.86-1.22) | 1.31 (1.10-1.56) | 1.47 (1.20-1.80) |
| **No intervention** |  |  |  |  |  |  |  |
| In-Hospital Mortality^1^ | 711 (2.8%)  (2.6%-3.0%) | 1,946 (3.0%)  (2.9%-3.1%) | 2,129 (3.7%)  (3.6%-3.9%) | 1,564 (4.9%)  (4.7%-5.2%) | 1.09 (1.08-1.09) | 1.44 (1.35-1.37) | 1.93 (1.81-1.83) |
| 30-day all-cause mortality | 1,483 (5.8%)  (5.5%-6.1%) | 4,808 (7.5%)  (7.3%-7.7%) | 5,564 (9.8%)  (9.5%-10.0%) | 4,768 (15.1%)  (14.7%-15.5%) | 1.29 (1.22-1.37) | 1.72 (1.62-1.82) | 2.72 (2.57-2.89) |
| One-year all-cause mortality | 4,683 (20.4%)  (19.9%-20.9%) | 13,136 (22.1%)  (21.8%-22.4%) | 14,714 (27.9%)  (27.6%-28.3%) | 12,119 (41.3%)  (40.7%-41.9%) | 1.13 (1.09-1.17) | 1.48 (1.43-1.53) | 2.37 (2.29-2.45) |
| 30-day perioperative safety event^2^ | 2,231 (8.7%)  (8.3%-9.0%) | 5,534 (8.5%)  (8.3%-8.8%) | 5,158 (9.0%)  (8.8%-9.3%) | 2,633 (8.3%)  (8.0%-8.6%) | 0.99 (0.94-1.04) | 1.05 (1.00-1.10) | 0.97 (0.92-1.03) |

Cumulative incidences are presented as N (%) (95% CI)

^1^Odds ratios derived from logistic regression are reported for in-hospital mortality.

^2^Cause-specific hazard ratios are reported for 30-day perioperative safety events to account for the competing risk of death.

**Supplemental Table 3:** Cumulative incidence and adjusted hazard ratios for patients who underwent catheter-based interventions, stratified by sex.

| **Subject Characteristic** | **Male (N = 88,575)** | **Female (N = 106,311)** | **Adjusted HR**  **(Male vs. Female)** |
| --- | --- | --- | --- |
| **Catheter-Based Intervention** |  |  |  |
| In-Hospital Mortality^1^ | 255 (3.6%)  (3.2%-4.1%) | 304 (3.9%)  (3.5%-4.4%) | 0.92 (0.78-1.09) |
| 30-day all-cause mortality | 444 (6.36%)  (5.78%-6.93%) | 512 (6.67%)  (6.11%-7.23%) | 0.95 (0.84-1.08) |
| One-year all-cause mortality | 1,119 (17.84%)  (16.88%-18.81%) | 1,239 (17.91%)  (17.00%-18.83%) | 0.99 (0.91-1.07) |
| 30-day perioperative safety event^2^ | 699 (9.99%)  (9.30%-10.71%) | 757 (9.83%)  (9.18%-10.51%) | 1.02 (0.92-1.13) |
| **No intervention** |  |  |  |
| In-Hospital Mortality^1^ | 3,000 (3.7%)  (3.5%-3.8%) | 3,349 (3.4%)  (3.3%-3.5%) | 1.08 (1.02-1.13) |
| 30-day all-cause mortality | 7,585 (9.35%)  (9.15%-9.55%) | 9,037 (9.29%)  (9.11%-9.47%) | 1.01 (0.98-1.04) |
| One-year all-cause mortality | 20,303 (27.18%)  (26.86%-27.50%) | 24,348 (27.12%)  (26.82%-27.41%) | 1.00 (0.98-1.02) |
| 30-day perioperative safety event^2^ | 7,815 (9.57%)  (9.37%-9.78%) | 7,741 (7.92%)  (7.75%-8.09%) | 1.23 (1.19-1.27) |

Cumulative incidences are presented as N (%) (95% CI)

^1^Odds ratios derived from logistic regression are reported for in-hospital mortality.

^2^Cause-specific hazard ratios are reported for 30-day perioperative safety events to account for the competing risk of death.

**Supplemental Table 4:** Cumulative incidence and adjusted hazard ratios for patients who underwent catheter-based interventions, stratified by race.

| **Subject Characteristic** | **White (N = 160,966)** | **Asian (N = 1,249)** | **Black  (N = 25,255)** | **Other (N = 7,416)** | **Adjusted HR**  **(Asian vs White)** | **Adjusted HR (Black vs White)** | **Adjusted HR (Other vs White)** |
| --- | --- | --- | --- | --- | --- | --- | --- |
| **Catheter-Based Intervention** |  |  |  |  |  |  |  |
| In-Hospital Mortality^1^ | 443 (3.7%)  (3.3%-4.0%) | 1 (1.7%)  (0.1%-7.2%) | 101 (5.1%)  (4.2%-6.2%) | 13 (2.2%)  (1.2%-3.8%) | 0.45 (0.10-2.10) | 1.52 (0.30-6.57) | 0.65 (0.13-2.80) |
| 30-day all-cause mortality | 781 (6.5%)  (6.1%-6.9%) | 5 (5.6%)  (0.7%-10.5%) | 145 (7.3%)  (6.2%-8.5%) | 25 (4.4%)  (2.7%-6.1%) | 0.85 (0.35-2.06) | 1.14 (0.95-1.35) | 0.67 (0.45-0.99) |
| One-year all-cause mortality | 1914 (17.6%)  (16.9%-18.3%) | 22 (29.7%)  (19.0%-40.3%) | 345 (20.1%)  (18.1%-22.0%) | 77 (14.9%)  (11.8%-18.0%) | 1.75 (1.15-2.66) | 1.16 (1.03-1.29) | 0.84 (0.67-1.05) |
| 30-day perioperative safety event^2^ | 1187 (9.8%)  (9.3%-10.4%) | 3 (-) | 215 (10.9%)  (9.5%-12.2%) | 52 (9.1%)  (6.7%-11.4%) | 0.30 (0.09-0.98) | 1.11 (0.96-1.28) | 0.91 (0.69-1.20) |
| **No intervention** |  |  |  |  |  |  |  |
| In-Hospital Mortality^1^ | 5,071 (3.4%)  (3.3%-3.5%) | 59 (5.1%)  (3.9%-6.5%) | 974 (4.2%)  (3.9%-4.4%) | 246 (3.6%)  (3.2%-4.1%) | 1.53 (1.46-1.59) | 1.32 (1.18-1.29) | 1.13 (1.01-1.10) |
| 30-day all-cause mortality | 13,835 (9.4%)  (9.2%-9.5%) | 145 (12.7%)  (10.8%-14.6%) | 2,066 (8.9%)  (8.6%-9.3%) | 576 (8.5%)  (7.8%-9.2%) | 1.38 (1.17-1.63) | 0.95 (0.91-1.00) | 0.90 (0.83-0.98) |
| One-year all-cause mortality | 36,880 (27.1%)  (26.8%-27.3%) | 356 (34.7%)  (31.7%-37.7%) | 5,832 (27.8%)  (27.2%-28.4%) | 1,584 (25.6%)  (24.5%-26.7%) | 1.34 (1.21-1.49) | 1.02 (0.99-1.05) | 0.94 (0.89-0.98) |
| 30-day perioperative safety event^2^ | 12,412 (8.4%)  (8.2%-8.5%) | 123 (10.7%)  (8.9%-12.5%) | 2,375 (10.2%)  (9.8%-10.6%) | 647 (9.5%)  (8.8%-10.2%) | 1.30 (1.09-1.55) | 1.23 (1.18-1.28) | 1.13 (1.05-1.23) |

Cumulative incidences are presented as N (%) (95% CI)

^1^Odds ratios derived from logistic regression are reported for in-hospital mortality.

^2^Cause-specific hazard ratios are reported for 30-day perioperative safety events to account for the competing risk of death.

**Supplemental Table 5:** Cumulative incidence and adjusted hazard ratios for patients who underwent catheter-based interventions, stratified by geographic region.

| **Subject Characteristic** | **Northeast (N = 34,676)** | **Midwest (N = 29,061)** | **South (N = 97,785)** | **West (N = 33,364)** | **Adjusted HR (Midwest vs Northeast)** | **Adjusted HR (South vs Northeast)** | **Adjusted HR (West vs Northeast)** |
| --- | --- | --- | --- | --- | --- | --- | --- |
| **Catheter-Based Intervention** |  |  |  |  |  |  |  |
| In-Hospital Mortality^1^ | 109 (4.2%)  (3.5%-5.1%) | 72 (3.3%)  (2.6%-4.2%) | 257 (3.5%)  (3.1%-4.0%) | 120 (4.5%)  (3.7%-5.3%) | 0.79 (0.73-0.84) | 0.83 (0.77-0.88) | 1.07 (0.99-1.14) |
| 30-day all-cause mortality | 177 (6.9%)  (5.9%-7.9%) | 172 (8.0%)  (6.8%-9.1%) | 444 (6.1%)  (5.5%-6.6%) | 162 (6.1%)  (5.2%-7.0%) | 1.16 (0.94-1.42) | 0.88 (0.74-1.04) | 0.88 (0.71-1.09) |
| One-year all-cause mortality | 493 (19.5%)  (18.0%-21.1%) | 392 (18.5%)  (16.9%-20.2%) | 1,300 (18.2%)  (17.3%-19.1%) | 409 (15.5%)  (14.2%-16.9%) | 0.95 (0.83-1.08) | 0.92 (0.83-1.02) | 0.78 (0.69-0.89) |
| 30-day perioperative safety event^2^ | 225 (8.7%)  (7.6%-9.8%) | 211 (9.8%)  (8.5%-11.0%) | 747 (10.2%)  (9.5%-10.9%) | 274 (10.2%)  (9.1%-11.4%) | 1.13 (0.94-1.36) | 1.19 (1.02-1.37) | 1.19 (1.00-1.41) |
| **No intervention** |  |  |  |  |  |  |  |
| In-Hospital Mortality^1^ | 1,242 (3.9%)  (3.7%-4.1%) | 1,008 (3.8%)  (3.5%-4.0%) | 2,999 (3.3%)  (3.2%-3.4%) | 1,100 (3.6%)  (3.4%-3.8%) | 0.97 (0.96-0.97) | 0.92 (0.85-0.86) | 0.99 (0.91-0.92) |
| 30-day all-cause mortality | 3,022 (9.5%)  (9.2%-9.8%) | 2,501 (9.4%)  (9.0%-9.7%) | 8,328 (9.3%)  (9.1%-9.5%) | 2,772 (9.1%)  (8.7%-9.4%) | 0.99 (0.94-1.04) | 0.98 (0.94-1.02) | 0.95 (0.90-1.00) |
| One-year all-cause mortality | 8,112 (27.8%)  (27.2%-28.3%) | 6,710 (27.3%)  (26.7%-27.9%) | 22,645 (27.5%)  (27.1%-27.8%) | 7,185 (25.5%)  (24.9%-26.0%) | 0.98 (0.95-1.01) | 0.98 (0.96-1.01) | 0.90 (0.88-0.93) |
| 30-day perioperative safety event^2^ | 2,948 (9.2%)  (8.9%-9.6%) | 2,196 (8.2%)  (7.9%-8.5%) | 7,815 (8.7%)  (8.5%-8.9%) | 2,597 (8.4%)  (8.1%-8.8%) | 0.88 (0.84-0.93) | 0.94 (0.90-0.98) | 0.91 (0.86-0.96) |

Cumulative incidences are presented as N (%) (95% CI)

^1^Odds ratios derived from logistic regression are reported for in-hospital mortality.

^2^Cause-specific hazard ratios are reported for 30-day perioperative safety events to account for the competing risk of death.

**Supplemental Table 6:** Cumulative incidence and adjusted hazard ratios for patients who underwent catheter-based interventions, stratified by teaching hospital status.

| **Subject Characteristic** | **Teaching (N = 141,938)** | **Non-Teaching (N = 52,948)** | **Adjusted HR (Teaching vs. Non-Teaching)** |
| --- | --- | --- | --- |
| **Catheter-Based Intervention** |  |  |  |
| In-Hospital Mortality^1^ | 452 (4.2%)  (3.8%-4.5%) | 107 (2.8%)  (2.3%-3.3%) | 1.53 (1.23-1.89) |
| 30-day all-cause mortality | 705 (6.52%)  (6.06%-6.99%) | 250 (6.52%)  (5.74%-7.30%) | 1.01 (0.87-1.16) |
| One-year all-cause mortality | 1,776 (18.33%)  (17.55%-19.12%) | 581 (16.63%)  (15.38%-17.88%) | 1.10 (1.00-1.20) |
| 30-day perioperative safety event^2^ | 1,042 (9.60%)  (9.06%-10.17%) | 415 (10.77%)  (9.81%-11.77%) | 0.89 (0.79-0.99) |
| **No intervention** |  |  |  |
| In-Hospital Mortality^1^ | 5,009 (3.8%)  (3.7%-3.9%) | 1,341 (2.7%)  (2.6%-2.9%) | 1.41 (1.32-1.49) |
| 30-day all-cause mortality | 12,495 (9.61%)  (9.45%-9.77%) | 4,128 (8.53%)  (8.29%-8.78%) | 1.13 (1.09-1.17) |
| One-year all-cause mortality | 33,188 (27.66%)  (27.40%-27.92%) | 11,463 (25.76%)  (25.35%-26.18%) | 1.09 (1.07-1.12) |
| 30-day perioperative safety event^2^ | 11,584 (8.86%)  (8.71%-9.01%) | 3,972 (8.16%)  (7.92%-8.41%) | 1.09 (1.06-1.13) |

Cumulative incidences are presented as N (%) (95% CI)

^1^Odds ratios derived from logistic regression are reported for in-hospital mortality.

^2^Cause-specific hazard ratios are reported for 30-day perioperative safety events to account for the competing risk of death.

**Supplemental Table 7:** Cumulative incidence and adjusted hazard ratios for patients who underwent catheter-based interventions, stratified by distressed community status.

| **Subject Characteristic** | **Distressed (N = 39,398)** | **Non-distressed (N = 155,488)** | **Adjusted HR (Distressed vs. Non-distressed)** |
| --- | --- | --- | --- |
| **Catheter-Based Intervention** |  |  |  |
| In-Hospital Mortality^1^ | 97 (3.3%)  (2.7%-4.0%) | 462 (3.9%)  (3.6%-4.3%) | 0.83 (0.61-1.04) |
| 30-day all-cause mortality | 185 (6.32%)  (5.44%-7.20%) | 770 (6.57%)  (6.12%-7.02%) | 0.96 (0.82-1.12) |
| One-year all-cause mortality | 492 (18.79%)  (17.27%-20.31%) | 1,866 (17.66%)  (16.92%-18.39%) | 1.06 (0.96-1.17) |
| 30-day perioperative safety event^2^ | 339 (11.50%)  (10.38%-12.68%) | 1,118 (9.51%)  (8.99%-10.05%) | 1.22 (1.08-1.38) |
| **No intervention** |  |  |  |
| In-Hospital Mortality^1^ | 1,432 (3.9%)  (3.7%-4.1%) | 4,917 (3.4%)  (3.3%-3.5%) | 1.16 (1.09-1.23) |
| 30-day all-cause mortality | 3,486 (9.67%)  (9.36%-9.98%) | 13,137 (9.23%)  (9.08%-9.38%) | 1.05 (1.01-1.09) |
| One-year all-cause mortality | 9,592 (29.06%)  (28.56%-29.56%) | 35,059 (26.67%)  (26.42%-26.91%) | 1.10 (1.07-1.12) |
| 30-day perioperative safety event^2^ | 3,326 (9.17%)  (8.88%-9.47%) | 12,231 (8.54%)  (8.40%-8.69%) | 1.08 (1.04-1.12) |

Cumulative incidences are presented as N (%) (95% CI)

^1^Odds ratios derived from logistic regression are reported for in-hospital mortality.

^2^Cause-specific hazard ratios are reported for 30-day perioperative safety events to account for the competing risk of death.

**Supplemental Table 8:** Cumulative incidence and adjusted hazard ratios for patients who underwent catheter-based interventions, stratified by rural hospital status.

| **Subject Characteristic** | **Rural (N = 8,057)** | **Urban (N = 186,829)** | **Adjusted HR (Rural vs. Urban)** |
| --- | --- | --- | --- |
| **Catheter-Based Intervention** |  |  |  |
| In-Hospital Mortality^1^ | 21 (3.5%)  (2.2%-5.4%) | 538 (3.8%)  (3.5%-4.1%) | 0.93 (0.51-1.46) |
| 30-day all-cause mortality | 49 (8.41%)  (6.14%-10.68%) | 907 (6.44%)  (6.04%-6.85%) | 1.32 (0.99-1.75) |
| One-year all-cause mortality | 77 (14.28%)  (11.28%-17.28%) | 2,281 (18.03%)  (17.35%-18.71%) | 0.82 (0.65-1.02) |
| 30-day perioperative safety event^2^ | 66 (11.34%)  (8.91%-14.08%) | 1,391 (9.85%)  (9.37%-10.35%) | 1.16 (0.91-1.49) |
| **No intervention** |  |  |  |
| In-Hospital Mortality^1^ | 249 (3.3%)  (2.9%-3.8%) | 6,100 (3.5%)  (3.4%-3.6%) | 0.94 (0.83-1.07) |
| 30-day all-cause mortality | 664 (8.98%)  (8.33%-9.63%) | 15,959 (9.33%)  (9.19%-9.47%) | 0.96 (0.89-1.04) |
| One-year all-cause mortality | 1,746 (25.54%)  (24.49%-26.58%) | 42,905 (27.22%)  (26.99%-27.44%) | 0.93 (0.89-0.98) |
| 30-day perioperative safety event^2^ | 622 (8.38%)  (7.76%-9.02%) | 14,934 (8.68%)  (8.55%-8.82%) | 0.97 (0.89-1.05) |

Cumulative incidences are presented as N (%) (95% CI)

^1^Odds ratios derived from logistic regression are reported for in-hospital mortality.

^2^Cause-specific hazard ratios are reported for 30-day perioperative safety events to account for the competing risk of death.


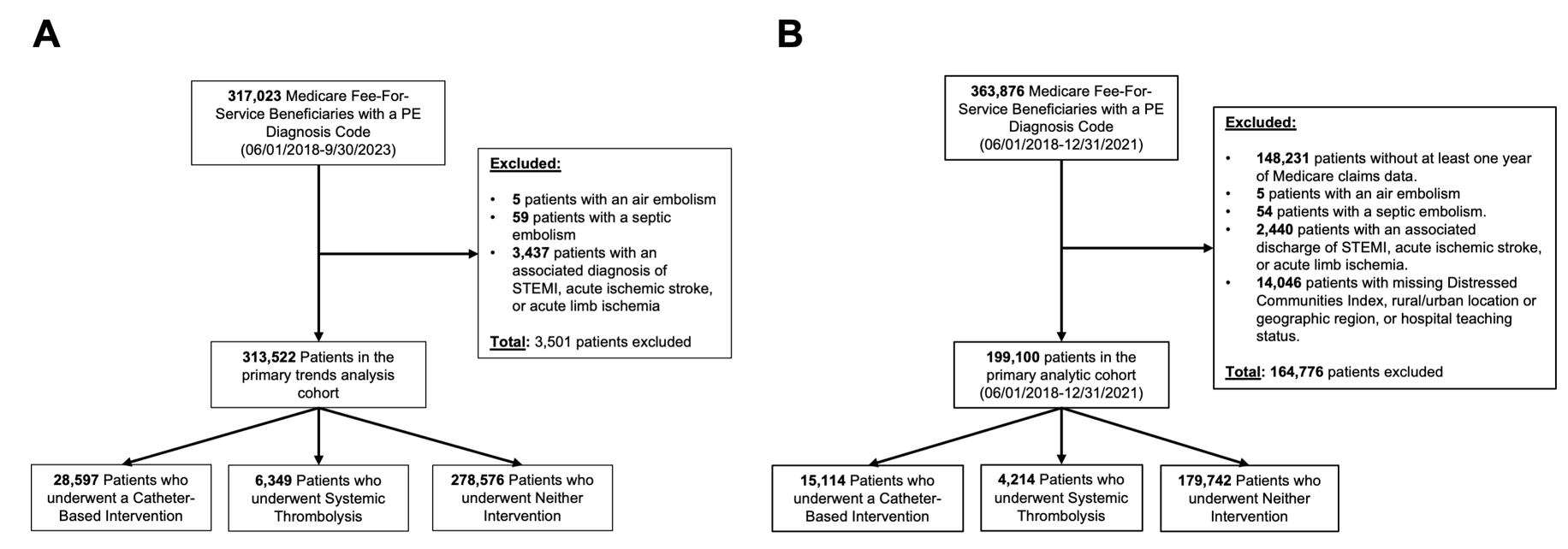


**Supplemental Figure 1:** Study flow diagram for cohort selection. Selection of **(A)** trends analysis cohort (06/01/2018-9/30/2023) and **(B)** outcomes analysis cohort (06/01/2018-12/31/2021).


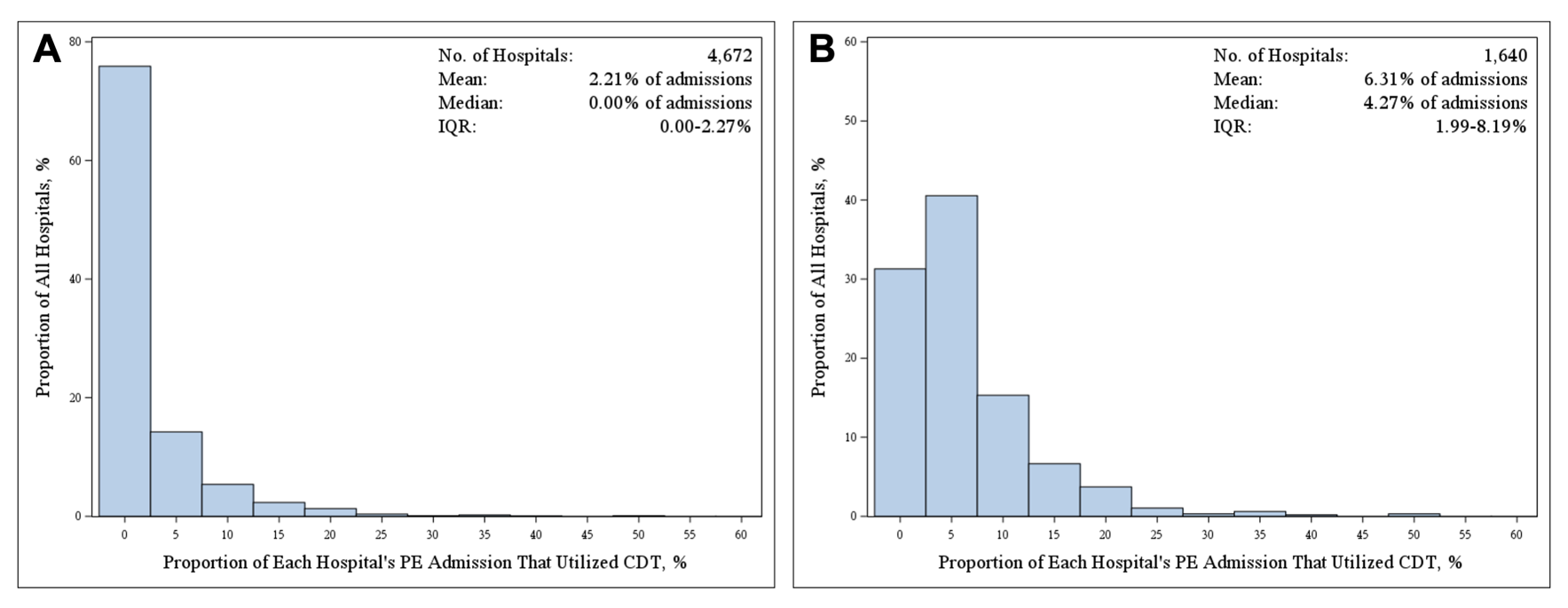


**Supplemental Figure 2:** Hospital variation in the utilization of catheter-directed thrombolysis during PE hospitalizations for **(A)** all hospitals and **(B)** hospitals who utilized at least one catheter-directed thrombolysis procedure.


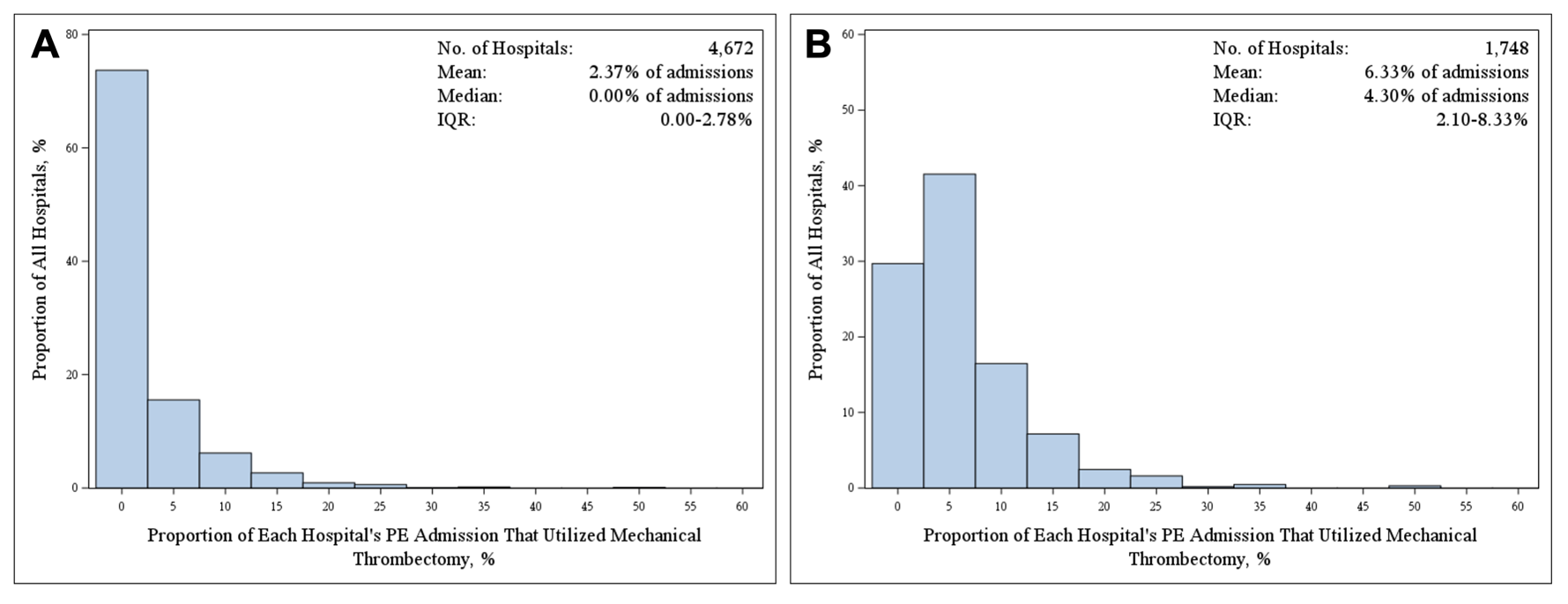


**Supplemental Figure 3:** Hospital variation in the utilization of mechanical thrombectomy during PE hospitalizations for **(A)** all hospitals and **(B)** hospitals who utilized at least one mechanical thrombectomy procedure.


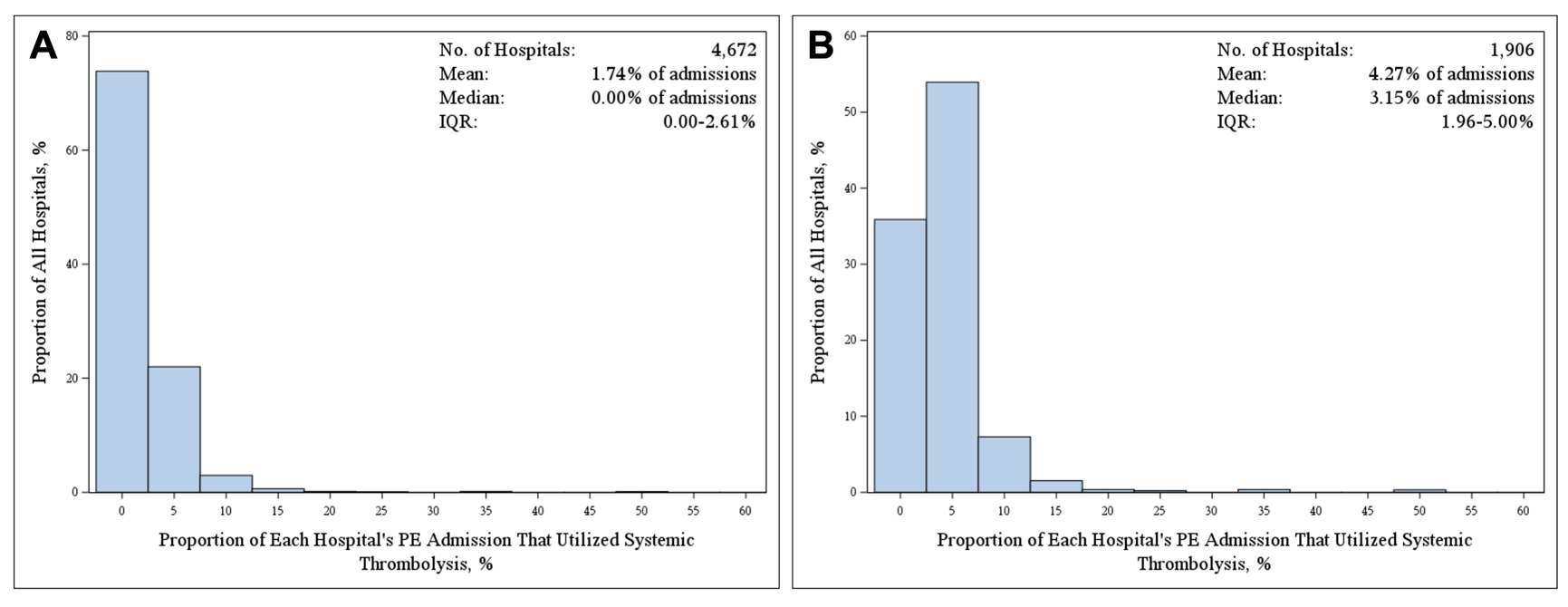


**Supplemental Figure 4:** Hospital variation in the utilization of systemic thrombolysis during PE hospitalizations for **(A)** all hospitals and **(B)** hospitals who utilized at least one systemic thrombolysis procedure.

**
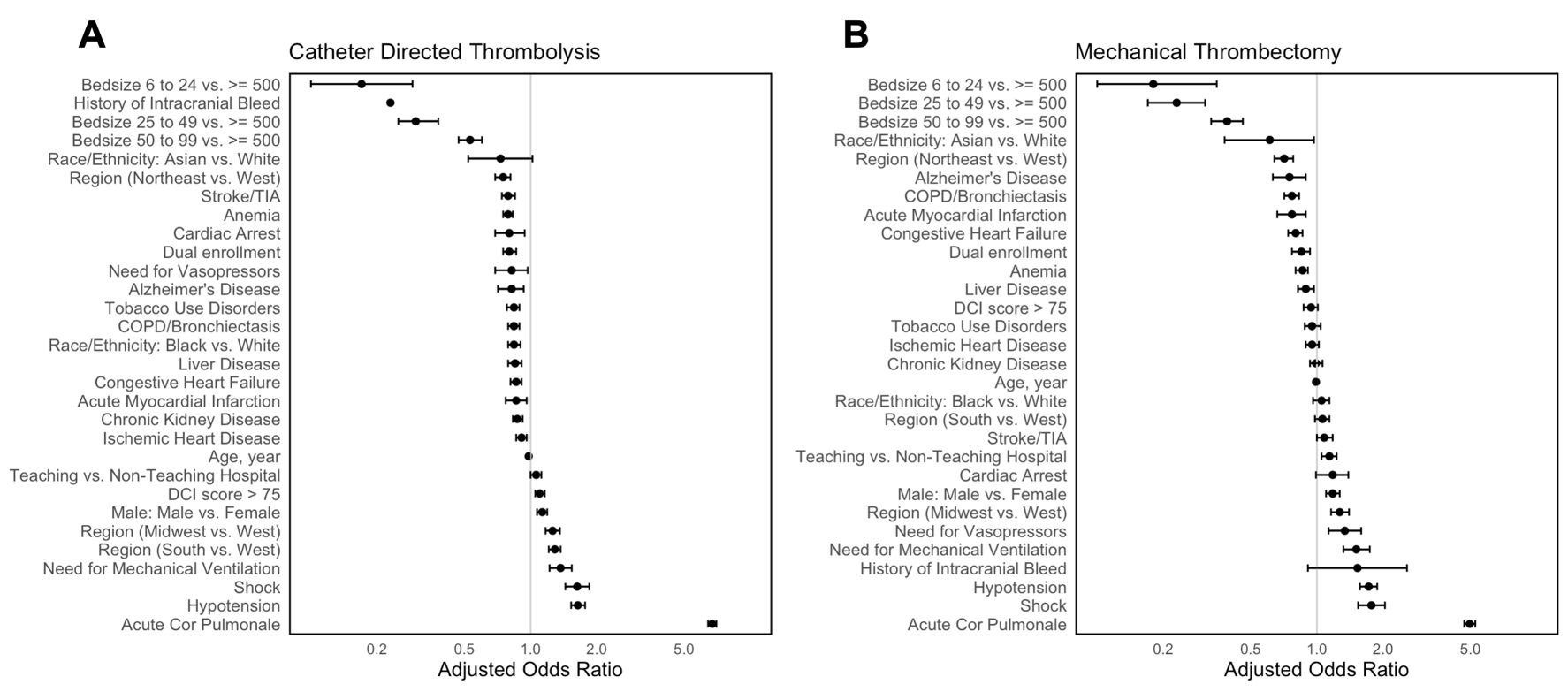
**

**Supplemental Figure 5:** Selected characteristics associated with the utilization of **(A)** catheter-directed thrombolysis and **(B)** mechanical thrombectomy.
